# Lipid–Polymer Hybrids Encapsulating Iron-Oxide Nanoparticles as a Label for Lateral Flow Immunoassays

**DOI:** 10.3390/bios11070218

**Published:** 2021-07-01

**Authors:** Shayesteh Bazsefidpar, Amanda Moyano, Gemma Gutiérrez, María Matos, María Carmen Blanco-López

**Affiliations:** 1 Department of Physical and Analytical Chemistry & Institute of Biotechnology of Asturias, University of Oviedo, c/Julián Clavería 8, 33006 Oviedo, Spain; bazsefidparshayesteh@uniovi.es (S.B.); moyanoamanda@uniovi.es (A.M.); 2 Department of Chemical and Environmental Engineering & Institute of Biotechnology of Asturias, University of Oviedo, 33006 Oviedo, Spain; gutierrezgemma@uniovi.es

**Keywords:** SPIONs, encapsulation, PLGA, lipid, lipid–polymer hybrid nanoparticles, lateral flow immunoassays (LFIA)

## Abstract

The feasibility of using Superparamagnetic Iron Oxide Nanoparticles (SPIONs) encapsulated by lipid–polymer nanoparticles as labels in lateral flow immunoassays (LFIA) was studied. First, nanoparticles were synthesized with average diameters between 4 and 7 (nm) through precipitation in W/O microemulsion and further encapsulated using lipid–polymer nanoparticles. Systems formulated were characterized in terms of size and shape by DLS (Nanozetasizer from Malvern) and TEM. After encapsulation, the average size was around (≈20 and 50 nm). These controlled size agglomerates were tested as labels with a model system based on the biotin–neutravidin interaction. For this purpose, the encapsulated nanoparticles were conjugated to neutravidin using the carbodiimide chemistry, and the LFIA was carried out with a biotin test line. The encapsulated SPIONs showed that they could be promising candidates as labels in LFIA test. They would be useful for immunomagnetic separations, that could improve the limits of detection by means of preconcentration.

## 1. Introduction

Point-of-care (POC) tests are diagnostic tools for rapid detection and fast analysis [1,2]. Lateral flow immunoassays are among the most popular devices for POC diagnostics [3,4]. Lateral flow immunoassays (LFIA), also called immunochromatographic tests, have been established as POC tests in recent years due to the rapid, low-cost, simple detection [5]. They are the basis for the well-known pregnancy test, but they can be designed for different applications in biomedicine, toxicology, food, agriculture, and environmental fields [6,7]. They consist of a membrane of nitrocellulose, where bioreceptors are immobilized. As the sample flows through the membrane, the analyte is captured at the test line. The molecular recognition event is revealed by means of labeled bioreagents. For the actual pandemic situation, LFIA tests are attracting a lot of interest because they are the basis of antigen and antibody tests for COVID-19 diagnostics [8].

However, their usage is still limited due to the poor sensitivity, specificity, and limited stability [3,9]. The actual challenges include the need to achieve low limits of detection, with direct use or simplified protocols of sample preparation, or the possibility for quantification. The nanopaticles used in LFIAs play an important role in the sensitivity of the system [5]. The most common are gold or latex nanoparticles, which are good for visual detection. In recent years, nanoparticle research has been a cutting edge field in biosensing, and other particles with fluorescent, magnetic or electrical properties are currently investigated in this field [1,10,11]. Among nanoparticles, Superparamagnetic Iron Oxide Nanoparticles (SPIONs) have received considerable attention based on their properties such as potent magnetic, superparamagnetic properties, high surface-area-to-volume ratio, and quick magnetophoretic response for the development of a new generation of LFIA [11,12,13]. However, SPIONs have several limitations such as aggregation, lack of normal distribution in the suspension in water, and lack of maintenance of the long-term stability of functionalized SPIONs which limit their usage in point-of-care diagnosis [1]. Therefore, encapsulation of SPIONs could be one of the most useful methods for controlled agglomeration of nanoparticles, improving the stability of the suspension in water, preventing oxidation, reducing the toxicity of nanoparticles, and facilitating the bioconjugation of nanoparticles with biological molecules [14,15,16].

Lipid–polymer hybrid nanoparticles (LPHNPs) are hybrid systems conceptually made of both liposomes and polymeric nanoparticles. In this system, polymer as a core is covered by a lipid layer. This system strongly enhances the encapsulation efficiency [17,18]. Encapsulation by LPHNPs offers several advantages such as enhancing device sensitivity as well as the possibility to modify the properties of the surface to provide a better interaction with biological molecules [19]. Poly (lactic-co-glycolic acid) (PLGA) presents a promising alternative to other conventional nanocolloids, such as nanoemulsions or liposomes. PLGA is one of the most successfully developed biodegradable hydrophobic polymers and hence presents biodegradability and biocompatibility. Moreover, it offers not only the possibility to encapsulate various types of drugs e.g., hydrophilic or hydrophobic small molecules or macromolecules, but also allows the possibility to modify surface properties to provide stealthiness and/or better interaction with biological materials [18,19]. Phosphatidylcholine (PC) is a neutral phospholipid that adsorbs and self-assembles onto the surface of the hydrophobic polymer through hydrophobic interactions with the goal of reducing the free energy of the system [18].

In this study, SPIONs through precipitation in W/O microemulsion were synthesized and further encapsulated in lipid–polymer hybrid system as labels for LFIA. Biodegradable PLGA used as a polymer core for entrapping SPIONs, PVA as a non-ionic surfactant for the stabilization, and PC as the lipid layer surrounding the polymer core (SPIONs in PLGA–PVA/PC). The emulsion/solvent method was used for the encapsulation of SPIONs in PLGA and PC. To test their application in LFIA, we studied these encapsulated SPIONs in PLGA–PVA/PC as labels in LFIA. The encapsulated SPIONs in PLGA–PVA/PC were bioconjugated to neutravidin and tested against biotin in the strips.

## 2. Materials and Methods

### 2.1. Chemicals

Ferric Chloride Hexahydrate (FeCl_3_∙6H_2_O) and (FeCl_2_∙4H_2_O) was supplied from Sigma-Aldrich (Madrid, Spain). Ammonia 30% (*v*/*v*) (NH_3_) were supplied by Panreac AppliChem (Barcelona, Spain). Ferrous Chloride Tetrahydrate. Cetyl Trimethyl Ammonium Bromide 99% (CTAB), 1-butanol (min. 99%) and hydrochloric acid 38% (HCl), ethanol (95%), and phosphotungstic acid hydrate (99.995%), 1-Hexanol, and Sodium Hydroxide (NaOH) were supplied by Sigma-Aldrich (Madrid, Spain). Nitric Acid, min. 69.5% (HNO_3_) was supplied by Scharlau (Barcelona, Spain).

PLGA (LG 50:50, Mw 24–38 kDa) was purchased from Sigma Chemical Co. (Steinheim, Germany). Polyvinyl alcohol (PVA) (Mw 30–70 kDa), phosphatidylcholine (PC) (predominant species: C42H80NO8P, MW = 775.04 g/mol) from soybean (Phospholipon 90G) was obtained from Lipoid (Koln, Germany). Cholesteryl hemisuccinate (MW = 486.73 g/mol) was obtained from Sigma Chemical Co. Sepharose^TM^ CL-4B was purchased from GE Healthcare company in (Stockholm, Sweden).

N-Hydroxysuccinimide (NHS), 1-ethyl-3-[3-dimethylaminopropyl]–carbodiimide hydrochloride (EDC), 2-(N-morpholino) ethanesulfonic acid (MES), bovine serum albumin (BSA) were purchased by Sigma-Aldrich. Neutravidin protein was provided from Thermo Fischer Scientific (Waltham, MA, USA).

For biotin–neutravidin tests, glass fiber membrane (GFCP001000) was employed as sample pad and backing cards (HF000MC100) were obtained from Millipore (Darmstadt, Germany). Other materials used were nitrocellulose membranes (UniSart CN95, Sartorius, Spain) and absorbent pads (Whatman, Madrid, Spain). 4-(2-hydroxyethyl)-1-piperazineethanesulfonic acid (HEPES) was used as a buffer for conjugation. The sample buffer consisted of 10 mM phosphate buffer (PB) pH 7.4 with 0.5% Tween-20 and 1% BSA.

### 2.2. Synthesis and Characterization of SPIONs

#### 2.2.1. Synthesis of SPIONs

SPIONs were produced based on two different formulations following the *W*/*O* microemulsion method reported in previous studies [20]. *W*/*O* microemulsions were formulated using CTAB as the main surfactant, 1-butanol as a cosurfactant, and 1-hexanol as the continuous oily phase. CTAB and 1-butanol were used using a constant weight ratio of 3:2 (CTAB: 1-butanol). The water phase consisted of a solution that contained Fe^2+^/Fe^3+^ in a 2:1 molar ratio, being 0.7 and 1.4 M, respectively. It was prepared by dissolving an appropriate amount of the chloride salts aforementioned being homogenized by magnetic stirring. The 0.01 M HCl solution was added to avoid Fe (II) oxidation. Both the formulation of the *W*/*O* microemulsions used for the synthesis of the SPIONs and the resulting mean diameters are shown in Table 1.

First of all, the microemulsions were prepared and left to rest for a while until the appearance was totally translucid. Then, the synthesis of the SPIONs was performed through the co-precipitation of the iron salts present in the microemulsion water droplets by the dropwise addition of ammonia (30% (*v*/*v*) solution) upon vigorous stirring with the Silent Crusher M Homogenizer (Heidolph 8F) (Atlanta, GA, USA) (set at 6500 rpm. Once a black precipitate appeared, the solution was left for two hours under magnetic stirring. Finally, all the samples were washed five times using a solution consisting of ethanol and water in a ratio of 90:10 (% *v*/*v*) and dispersed in ethanol or in water.

#### 2.2.2. Preparation of Hybrid Nanoparticles Encapsulating SPIONs

SPIONs were encapsulated by a single emulsion/solvent method. The two formulations of SPIONs synthesized (1 and 2) were previously dispersed in water or ethanol and therefore 4 sets of experiments were carried out.

For encapsulation, 400 μL of SPIONs dispersed (in ethanol or water) were added into 2 mL of organic phase consisting of 12.5% (*v*/*v*) methanol in chloroform solution. Then, 30 mg of PLGA and cholesteryl hemisuccinate 1% (*w*/*v*) of the total membrane compounds (PC) was dissolved in the solution. To prepare the hybrid nanoparticles (SPIONs in PLGA–PVA/PC), the organic solution was emulsified in 6 mL of aqueous phase containing 10 mg PC and 2% PVA (*w*/*v*) to form an oil-in-water (*O*/*W*) emulsion under continuous sonication with the amplitude of 70% for 5 min on an ice bath. After sonication, 8 mL of PVA 5% (*w*/*v*) were added and the sonication continued for 5 min. Then, the emulsions were stirred using a mechanical stirrer (Teflon type) (Fenteer Brand in Shenzhen, China) to allow evaporation of the organic solvent and form the particles for 24 h. Nanoparticles were purified using Size Exclusion Chromatography (SEC) including 13.5 cc of Sepharose^TM^ CL-4B and 20 cc of milli-Q water.

### 2.3. Bioconjugation of Lipid–Polymer SPIONs

Encapsulated SPIONs with carboxyl functional groups were functionalized using neutravidin to test their function as the label in LFIA through the interaction of neutravidin–biotin. Firstly, the carboxyl groups of the nanoparticles were activated using carbodiimide chemistry. For this, 0.0015 g of EDC and 0.0030 g of NHS were dissolved in 100 μL of MES buffer (pH 5.73) and were mixed with 1000 μL of nanoparticles for 30 min. After shaking, nanoparticles were separated from supernatant through a centrifuge at 5000× *g* for 4 min and washed with 200 μL of MES buffer (pH 5.73). Then, 100 μL of MES buffer (pH 7.4) and 100 μL of neutravidin (1 mg/mL) were added to nanoparticles. After shaking for 3 h, the excess EDC and NHS were removed via centrifuge at 5000× *g* for 4 min and washed with 200 μL of MES buffer (pH 7.4). In the next step, the residual carboxyl groups on the surfaces were blocked by adding 100 μL of MES buffer (pH 7.4) and 100 μL of BSA (0.010 g/L mL MES buffer (pH 7.4)) and reacted through shaking for 30 min. Then, the mixture was centrifuged at 5000× *g* for 4 min and washed with MES buffer (pH 7.4)). Finally, the supernatant was discarded, and nanoparticles were dispersed in 100 μL of PVA and PB buffer separately for comparison of their movement on the strips. After dispersing of nanoparticles 20 μL were added to 80 μL of running buffer containing Tween 20%, BSA, and PB buffer (pH = 7.4).

### 2.4. Characterization of Nanoparticles Conjugates

#### 2.4.1. Dynamic Light Scattering and Zeta Potential

Encapsulated SPIONs were characterized in terms of measurement of particle size distribution as well as zeta potential by dynamic light scattering (DLS) analysis using Zetasizer Nano ZS ZEN3600 (Malvern Instruments, Malvern, UK) equipped with a solid-state He–Ne laser (λ = 633 nm). Measurements were performed at 25 °C. Additionally, this instrument was used to monitor the conjugation process. Zetasizer software version 7.03 was used for data analysis.

#### 2.4.2. Transmission Electron Microscopy

Particle morphology, size, and the structure of aggregation of the encapsulated SPIONs were determined by TEM. The aqueous dispersion was drop-cased onto the former-coated copper grid and placed in the TEM for analysis with a JEOL-2000 Ex II TEM (Saint-Herblain, France).

### 2.5. Lateral Flow Assays

#### Preparation of the Strips

The LFIA was based on a dipstick format. The test strips were composed of a sample pad, nitrocellulose membrane, absorbent pad, and backing plastic card. Firstly, the nitrocellulose membrane (25 mm wide) was attached to a backing plastic card to get robustness. Then, for the test of biotin–neutravidin affinity, a test line of biotin-BSA was immobilized across the membrane by the IsoFlow dispenser (Imagene Technology, Lebanon, NH, USA) at a rate of 0.100 μL/mm. In the next step, the nitrocellulose membrane after the immobilization of the biotin-BSA test line was kept for 20 min at 37 °C. Finally, the sample pad and the absorbent pad were stuck onto the backing card with an overlap between them of 2 mm. The complete card was cut into 5 mm wide strips.

## 3. Results and Discussion

### 3.1. Characterization of the Lipid–Polymer SPIONs before Bioconjugation

**Figure 1:**
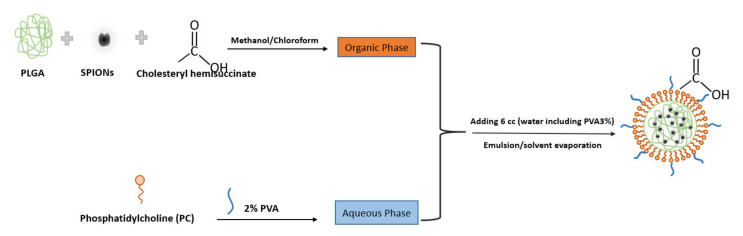
Scheme showing the preparation and structure of the SPIONs in PLGA–PVA/PC by emulsion/solvent evaporation method.

Figure 1 indicates the scheme of the preparation and structure of the SPIONs loading in lipid–polymer nanoparticle by emulsion/solvent method. In this procedure, PLGA was used as a carrier to entrap the nanoparticles and coat them with lipid layers. PVA (a non-ionic surfactant) was used as a stabilizer in the formulation.

**Figure 2:**
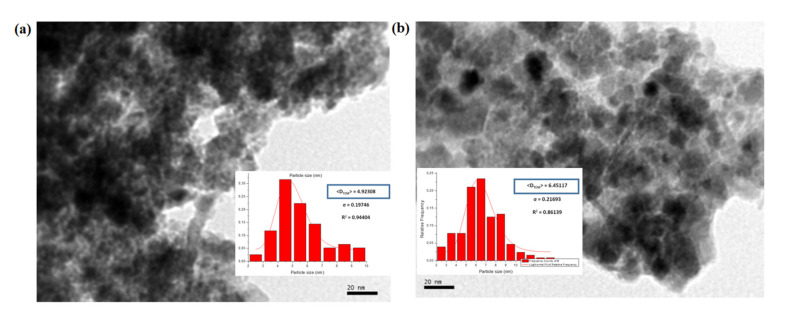

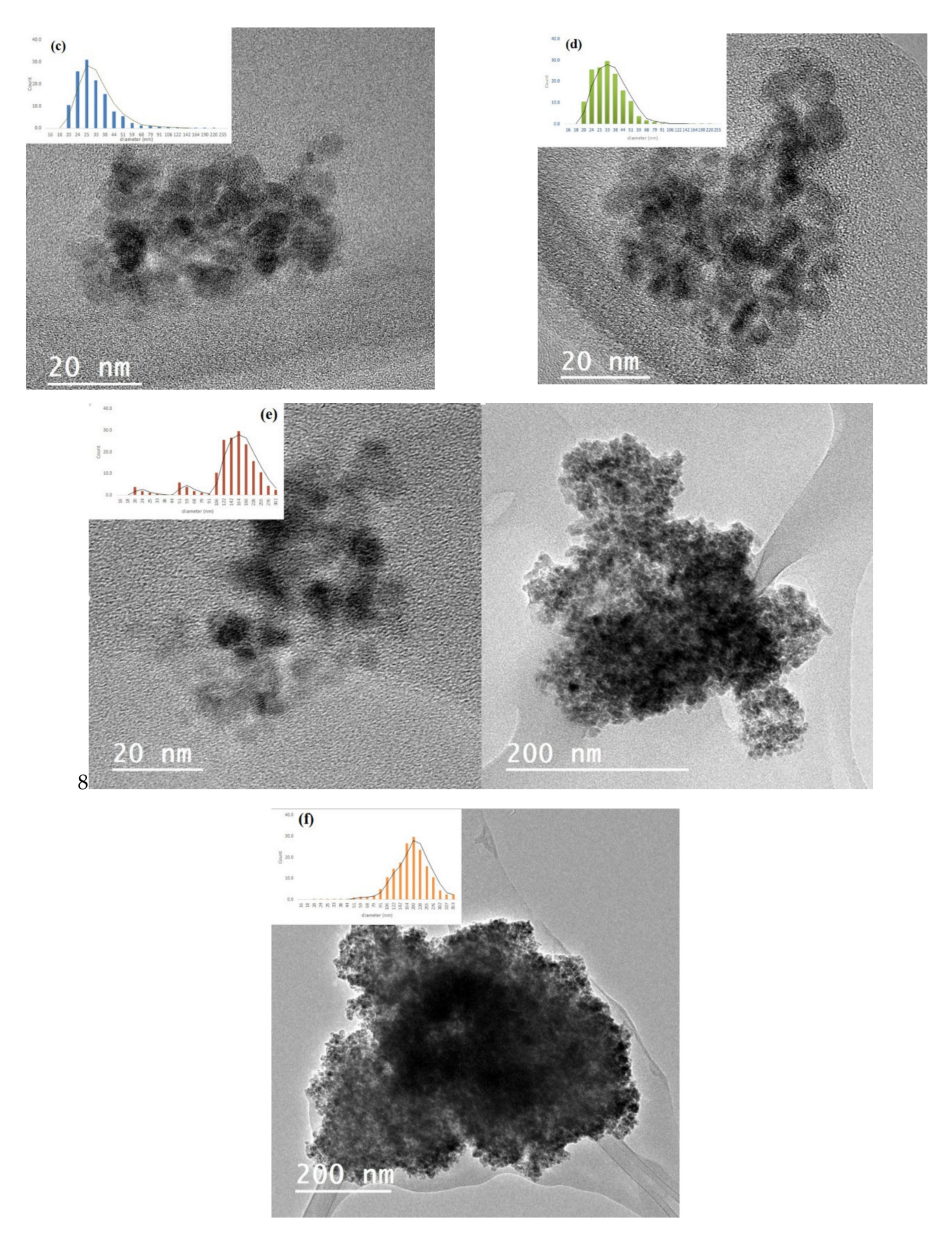
TEM images of the SPIONs in PLGA–PVA/PC. (**a**) SPIONs F1 before encapsulation (**b**) SPIONs F2 before encapsulation. (**c**) SPIONs F1 in the organic phase, (**d**) SPIONs F2 in the organic phase (**e**) SPIONs F1 in the water phase, (**f**) SPIONs F2 in the water phase.

Transmission electron microscopy (TEM) was used to investigate the state of aggregation of SPIONs in the lipid–polymer nanoparticles and to assess their size distribution. Figure 2a,b show that the SPIONs agglomerated under controlled size aggregates when they were dispersed in ethanol (organic phase). The average size of the agglomerates was around 20–50 nm, whereas that of single SPION was 4–7 nm. These micrographs show that the encapsulation of SPIONs in PLGA–PVA/PC was successful. However, when SPIONS were dispersed in the water phase, the encapsulation was not size-controlled (Figure 2c,d). The size obtained by DLS and zeta potential for SPIONs in PLGA–PVA/PC are summarized in Table 2. It was observed that after the addition of SPIONs, the hydrodynamic size of lipid–polymer nanoparticles was slightly larger than empty lipid–polymer nanoparticles (≈80 nm) in all samples. Additionally, the zeta potential of SPIONs in PLGA–PVA/PC nanoparticle was higher than empty lipid–polymer nanoparticles (≈−0.3 mV) that relied on the presence of cholesteryl hemisuccinate.

### 3.2. Biotin–Neutravidin Affinity Test

**Figure 3:**
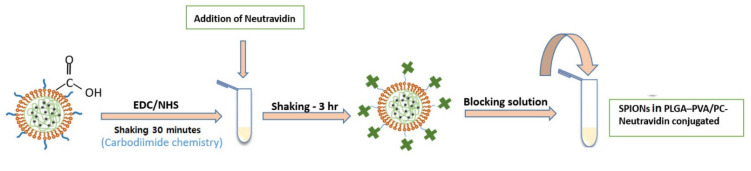
A schematic of the protocol of bioconjugation of encapsulated SPIONs with carboxyl functional groups with neutravidin.

The biotin–neutravidin complex is one of the strongest non-covalent interactions that due to the high specificity and strong affinity is widely used in immunoassays [21,22]. In this study, the biotin–neutravidin system was applied as model system in order to study the feasibility of using SPIONs in PLGA–PVA/PC as labels for LFIA. Figure 3 shows the steps of bioconjugation of encapsulated SPIONs with neutravidin.

After bioconjugation of the four different types of encapsulated SPIONs in PLGA–PVA/PC with neutravidin separately, bioconjugated nanoparticles were dispersed in PVA (1%, 3%, and 5%) and in PB separately to compare the movement of conjugated nanoparticles on the strips. The results showed that the bioconjugated nanoparticles dispersed in PVA 100 μL could flow through the strips, while the bioconjugated nanoparticles dispersed in PB did not. The movement of nanoparticles on strips in PVA 1% was weak but when using PVA 3% it was observed that nanoparticles moved better on the strips to make an optical signal, but it was not strong. The visual signal was optimized when using PVA 5% and this was the medium chosen for dispersing nanoparticles after bioconjugation.

**Figure 4:**
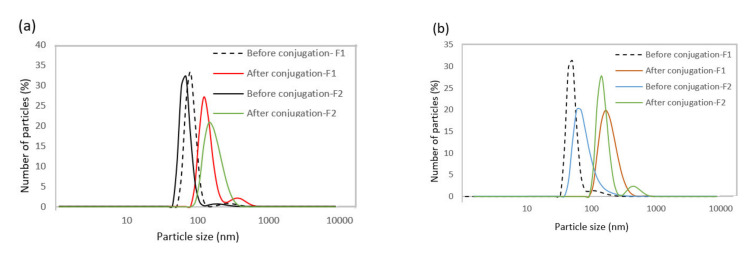
(**a**) Hydrodynamic diameter distribution profiles of SPIONs dispersed in the organic phase and (**b**) dispersed in the water phase before and after bioconjugation.

DLS was used to compare the hydrodynamic size of nanoparticles before and after the bioconjugation reaction (Table 3 and Figure 4). The results indicate that the hydrodynamic diameter of nanoparticles was higher after bioconjugation, confirming the success of this process.

**Figure 5:**
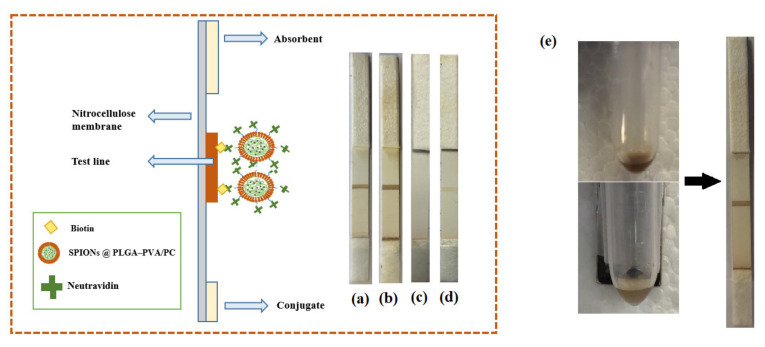
Scheme showing the preparation and structure of biotin–neutravidin affinity test. (**a**) SPIONs dispersed in the organic phase (F1), (**b**) SPIONs dispersed in the organic phase (F2), (**c**) SPIONs dispersed in the water phase (F1), (**d**) SPIONs disperses in the water phase (F2). (**e**) Separation of SPIONs in PLGA–PVA/PC nanoparticles using a conventional magnet.

In order to investigate the performance of four different formulations of encapsulated SPIONs for LFIA, 20 µL of suspensions and 80 µL of running buffer were transferred into a microtube. The strip was introduced into the microtube and the buffer including encapsulated SPIONs started to flow through the strips. As it can be seen in Figure 5, the SPIONs (1 and 2) dispersed in the organic phase could flow through the strips and make an optical signal in the test line. However, there was no signal when the SPIONs dispersed in the water phase were used. The unsuccessful encapsulation of SPIONs dispersed in the water phase in a controlled size could be the reason because they did not flow through the strips. In addition, encapsulated nanoparticles showed rapid separation with a conventional magnet in 1 min (Figure 5), whereas the free nanoparticles were very stable in colloidal suspension and they were not attracted by the magnet. On the other side, nanoparticles with larger size might not redisperse after removal of the magnetic field, but these lipid hybrids keep the ability to redisperse when the magnetic field is removed, and they have demonstrated good flowing behavior. This is very important for their successful use as labels when they are conjugated with the detection bioreagent.

**Figure 6:**
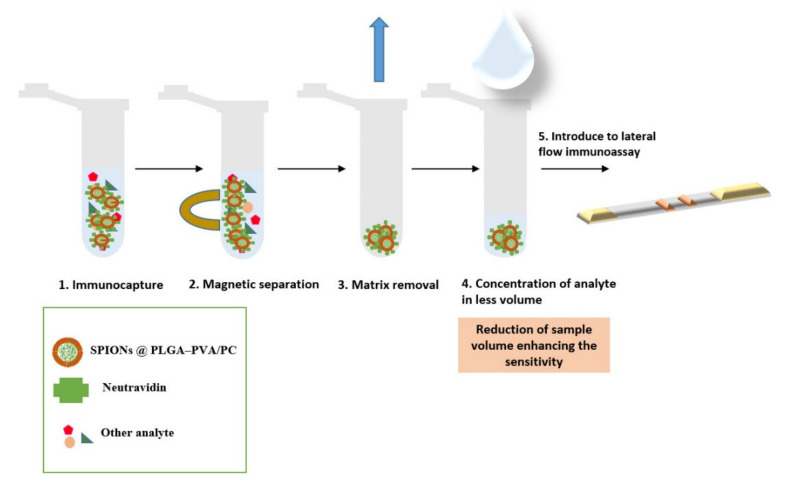
Scheme showing the immunomagnetic separation and preconcentration.

Figure 6 shows a scheme with the steps involved.

The intensity profiles of the nanoparticles at the biotin-BSA test line were measured for SPIONs in PLGA–PVA/PC and gold nanoparticles using ESEQuant Lateral Flow Reader (ESEQuant LRS). Table 4 shows the results obtained. It can be observed that the instrumental optical density reading of SPIONs in PLGA–PVA/PC nanoparticles was higher than that of gold nanoparticles (height and peak area).

This work shows proof of concept of the efficiency of the new labeling system and opens the path for applications in the health and environmental fields. The limits of detection could be greatly improved by using the capacity for magnetic separation of this lipid hybrid nanoparticle system.

## 4. Conclusions

In this work, the feasibility of using SPIONs encapsulated in lipid–polymer nanoparticles as labels for LFIA was investigated. SPIONs were synthesized with average diameters between 4 and 7 nm by precipitation using the W/O microemulsion method and were encapsulated in lipid–polymer nanoparticles by the emulsion/solvent method to obtain multinanoparticle systems based on SPIONs with controlled size. The results showed that SPIONs dispersed in the organic phase could be encapsulated with controlled size by lipid–polymer nanoparticles (SPIONsinPLGA–PVA/PC) providing an optical signal when using them as labels in lateral flow assays after bioconjugation with neutravidin. By using biotin-BSA test lines, this system has shown advantages such as good optical density intensity readings, as compared with gold nanoparticles. These multilabel systems have a great potential of application for magnetic separation and analyte preconcentration for these types of rapid tests.

## Figures and Tables

**Table 1:** W/O formulations used for the synthesis of SPIONs and mean sizes obtained by DLS.

Sample	Microemulsion Formulation (% *w*/*w*)				Size (nm)
	CTAB	1-Butanol	1-Hexanol	Water Phase	
1	24	16	45	15	5.4
2	15	10	57	18	6.6

**Table 2:** Results of the hydrodynamic size of SPIONs after encapsulation in lipid–polymer nanoparticles DLS (Z-average and polydispersity index).

Formulation	Size (nm)	PDI	Zeta Potential (mV)
Empty	80	0.243	−0.3
F1 (dispersed in organic phase)	82.34	0.263	−12.6
F2 (dispersed in organic phase)	89.45	0.345	−13.3
F1 (dispersed in water phase)	83.59	0.230	−13.43
F2 (dispersed in water phase)	99.87	0.229	−7.3

**Table 3:** DLS results of hydrodynamic diameter of SPIONs in PLGA–PVA/PC before and after conjugation with neutravidin.

Formulation	Size (nm) (Before Conjugation)	Size (nm) (After Conjugation)	PDI
F1 (dispersed in organic phase)	82.34	136.68	0.253
F2 (dispersed in organic phase)	89.45	150.35	0.225
F1 (dispersed in water phase)	83.59	164	0.230
F2 (dispersed in water phase)	99.87	142	0.234

**Table 4:** Comparison of the intensity profiles measured for gold nanoparticles and SPIONs in PLGA–PVA/PC nanoparticles.

Type of Nanoparticles	X-Pos (mm)	Intensity (mV)	Peak Start (mm)	Peak End (mm)	Height (mV)	Area (mm × mv)
SPIONs in PLGA–PVA/PC	25.48	1164	24.48	26.48	752.84	702.15
Gold	24.96	255	24.04	26.04	619.68	469.85

## Data Availability

Not applicable.
